# Molecular characterization and antifungal activity of lipopeptides produced from *Bacillus subtilis* against plant fungal pathogen *Alternaria alternata*

**DOI:** 10.1186/s12866-023-02922-w

**Published:** 2023-07-07

**Authors:** B. N. Harish, S. N. Nagesha, B. N. Ramesh, S. Shyamalamma, M. S. Nagaraj, H. C. Girish, C. Pradeep, K. S. Shiva Kumar, K. S. Tharun Kumar, S. N. Pavan, V. Kavan Kumar

**Affiliations:** 1 University of Agricultural Sciences, Bangalore, 560065 India; 2 ICAR-AICRP on Post Harvest Technology, UAS, GKVK, Bengaluru, 560065 India; 3 Indian Academy College, Bangalore, 560043 India; 4 Bangalore City College, Bangalore, 560043 India; 5 College of Technology and Engineering, MPUAT, Udaipur, Rajasthan 313001 India

**Keywords:** *B. subtilis*, Lipopeptides, HPLC, Antifungal activity, *Alternaria alternata*

## Abstract

Over 380 host plant species have been known to develop leaf spots as a result of the fungus *Alternaria alternata*. It is an aspiring pathogen that affects a variety of hosts and causes rots, blights, and leaf spots on different plant sections. In this investigation, the lipopeptides from the B. subtilis strains T3, T4, T5, and T6 were evaluated for their antifungal activities. In the genomic DNA, iturin, surfactin, and fengycin genes were found recovered from *B. subtilis* bacterium by PCR amplification. From different *B. subtilis* strains, antifungal Lipopeptides were extracted, identified by HPLC, and quantified with values for T3 (24 g/ml), T4 (32 g/ml), T5 (28 g/ml), and T6 (18 g/ml). To test the antifungal activity, the isolated lipopeptides from the B. subtilis T3, T4, T5, and T6 strains were applied to *Alternaria alternata* at a concentration of 10 g/ml. Lipopeptides were found to suppress *Alternaria alternata* at rates of T3 (75.14%), T4 (75.93%), T5 (80.40%), and T6 (85.88%). The T6 strain outperformed the other three by having the highest antifungal activity against *Alternaria alternata* (85.88%).

**Supplementary Information:**

The online version contains supplementary material available at 10.1186/s12866-023-02922-w.

## Introduction

The world population is increasing day by day, feeding this growing population was farmers’ main concern in the 1990s, but the issue has since been resolved by scientists and farmers through the development of a number of new agricultural techniques [1, 19]. However, the issues facing the globe today are of a healthy kind, such as issues with nutritious food, a healthy environment, and so forth. From the 1990s to the present, several sorts of research have been conducted in an effort to find safer agricultural inputs to replace the toxic chemicals used in agriculture and horticulture in order to achieve this healthy environment [4]. Scientists have discovered a number of biocontrol organisms. Today, pests, illnesses, and bacteria are managed by using fungi, bacteria, and insects as biocontrol agents [5, 6, 8]. The microflora and microfauna associated with crops that are typically injured by the chemicals that we use in modern agriculture are also saved by these biocontrol organisms, which substitute the chemicals in the field.

There are numerous biotic agents, including weeds, viruses, bacteria, nematodes, and insects, are encountered by agricultural crops [12]. By causing biotic stress, these agents interfere with the host crop’s natural metabolism. As a result, the crop’s growth and development are constrained, and the plant may even perish. However, not all biotic organisms are detrimental to the host plants; some of them are even helpful. Certain microorganisms are advantageous to the host plant and work as a biopesticide to conflict with pests and illnesses or as a biofertilizer to stimulate growth and development [9]. These interactions between the host plant and counter-biotic agent are symbiotic or synergistic [23, 24, 26]. Although a variety of bacteria are employed as biocontrol agents, the study is currently solely focused on plant growth-promoting rhizobacteria (PGPR) [14]. *B. subtilis* is a gram-positive, aerobic, rod-shaped, endospore-forming rhizobacterium that promotes plant development. It is an incredibly diversified bacterial species that can thrive in various environmental settings [2].

The spores have the ability to endure a variety of difficult stressful situations, and they can germinate when the environment is favourable. Numerous investigations have noted the lipopeptides from the bacteria *B. subtilis* broad antagonistic action against a variety of plant diseases [33], making them suitable biocontrol agents [19]. The lipopeptides found in *B. subtilis* are divided into three groups based on their structural relationships: the surfactin group, the fengycin group, and the iturin group [27], which are all amphiphilic membrane-active peptide antibiotics with strong antimicrobial properties and can be used as biopesticides to protect plants [15, 28]. A Gram-positive, catalase-positive bacteria called *B. subtilis*, sometimes referred to as the hay *Bacillus or grass Bacillus,* is found in soil at the ruminant and human digestive tracts [3].

*B. subtilis* is able to produce a hard, protective endospore that gives it the ability to withstand harsh environmental conditions. The most well-studied bacterium, *B. subtilis*, serves as a template for analysis of chromosome replication and bacterial cell growth. Christian Gottfried Ehrenberg gave it the original name *Vibrio subtilis*, and Ferdinand Cohn changed it to *B. subtilis* in 1872 [22]. *B. subtilis* can inhibit a variety of crucial plant diseases for agriculture, including *Fusarium sp*. [7, 35], *Rhizoctonia solani* [19], *Sclerotium rolfsii* [10], *Sporisorium reilianum* [25] and *Verticillium dahliae* [22]. In addition to controlling diseases, *B. subtilis* application can improve plant yields and growth [25]. *B. subtilis* play a significant role in improving tolerance to biotic stresses [17]. The expression of particular genes and hormones, such as 1-aminocyclopropane-1-carboxylate deaminase (ACC), is required for the induction of disease resistance. Ethylene controls root and shoot expansion and aids in preserving plant homeostasis. Under stressful circumstances, the breakdown of the ethylene precursor (ACC) by bacteria aids in relieving plant stress and maintaining normal growth [13]. Some of the volatile organic compounds (VOCs) produced by the *B. subtilis* strain (GB03) also help plants to resist pathogen attacks [18, 31]. *Bacillus spp*, also secrete exo-polysaccharides and siderophores that inhibit the movement of toxic ions and help to maintain the ionic balance, promote the movement of water in plant tissues, and inhibit the growth of pathogenic microbes [30]. The interaction of *B. subtilis* with host plants in the rhizosphere through root colonization, their biocontrol potential and mechanism of biocontrol, and the utilization of *B. subtilis* to maintain or increase crop productivity in the field under conditions of biotic and abiotic stress. Keeping all the above information in line and the present study was conducted with the objectives of molecular confirmation and in vitro bioassay of lipopeptides extracted from *B. subtilis* against *Alternaria alternata*.

## Materials and methods

### Experimental material

The various bacterial strains used, DNA isolation, PCR amplification, and anti-fungal activity of the *B. subtilis* are discussed in the following subheadings.

#### Bacterial strains

The Bacterial strains used were, T3 strain was sourced from Microbial Type Culture Collection (MTCC), T4, T5, and T6 strains were isolated from the Western Ghats of Karnataka Sakaleshpur, India as mentioned in Table 1. All *B. subtilis* strains were cultured in a 30 °C incubator with shaking at 180–200 rpm on Nutrient Agar (NA), Luria Bertaini (LB) broth, and Bacillus Differentiation Agar plates in order to create the ideal conditions for bacterial growth. Additionally, all *B. subtilis *strains were kept as glycerol stocks. New *B. subtilis* strains were cultivated for 24 h in LB medium before being transferred to cryovials and kept at -80 °C. A final concentration of 20% sterile glycerol was then added.

**Table 1:** *Bacillus subtilis* strains used in this study

**Sl. no**	**Bacterial stains**	**Strain id**
1	*Bacillus subtilis*	MTCC (T3)
2	*Bacillus subtilis*	HSN33 (T4)
3	*Bacillus subtilis*	HSNS48 (T5)
4	*Bacillus subtilis*	BJP3 (T6)

Source: The Microbial Type Culture Collection and Gene Bank (MTCC) used as reference

### Isolation of *B. subtilis* strains

A loop’s worth of soil was suspended in one or two drops of sterile water in a microfuge tube. For 10 min, the mixture was thoroughly mixed and heated at 80 degrees Celsius to kill the majority of gramme positive and gramme negative bacteria. After cooling the heat-treated soil samples were streaked on to nutrient agar plates using inoculation loop. 1–2 days of incubation at 30 °C, during which time several colonies on nutrient agar media were discovered. Colonies that were white, dry, or pasty in appearance were picked up and re-streaked on Bacillus differentiation Agar media, where they were cultured for 1–2 days at 30 °C. The entire collection of yellow colonies seen on Bacillus differentiation agar is isolated and streaked onto a master plate. To keep the culture pure and prevent contamination, subculture once every two weeks. 100 ml of LB broth contained injected Bacillus colonies, which were then incubated at 30 °C at 180–200 rpm [21]. All of the colonies were simultaneously checked for spore release. A cryogenic vial containing 1 ml of overnight-grown cultures was filled with 50% and 60% glycerol, vortexed, and kept at -80 °C.

### Quantification of DNA

#### DNA isolation

DNA was isolated using HiMedia—HiPurA™ Genomic DNA Purification Kit as per the manufacturers protocol. For Gram Positive bacterial preparation, a 45 mg/ml stock solution of lysozyme was prepared as described under general preparation instructions. Different strains of genomic DNA were detected using PCR. At Eurofins Scientific, Inc., oligonucleotides for PCR were created. Freeze-dried samples were dissolved in deionized water and kept at -20 °C. The primer sequences were synthesized are shown in the Table 2.

**Table 2:** Primers synthesized for the PCR amplification

Sl.no	**Oligo name**	**Sequence 5, to 3**^**,**^	**PCR Product size expected/Detected**	**Reference**
1	FENA - FP	CCCATCCGACYGTAGAAG	820	Mora et al. (2011) [27]
	FENA - RP	GTGTAAGCRGCAAGYAGCAC		
2	FENB - FP	CCTGGAGAAAGAATATACCGTACCY	670	
	FENB - RP	GCTGGTTCAGTIKGATCACAT		
3	FEND - FP	GGCCCGTTCTCTAAATCCAT	269	
	FEND - RP	GTCATGCTGACGAGAGCAAA		
4	SRFA - FP	TCGGGACAGGAAGACATCAT	201	
	SRFA - RP	CCACTCAAACGGATAATCCTGA		
5	ITUC - FP	CCCCCTCGGTCAAGTGAATA	594	Chung et al. (2008) [9]
	ITUC - RP	TTGGTTAAGCCCTGATGCTC		

### PCR amplification

#### PCR reaction

Bacterial genomic DNA was used to amplify the Iturin C*,* fen A*,* fen B*,* fen D, srf A genes. 1.25 U/μ of *Taq* DNA polymerase (Thermo Scientific, 5U/μl), 2 mM dNTPs each, 1 μM both primers and 3 mM of MgCl_2_ to the final volume 20 μl. Amplification was carried out using an Eppendorf thermocycler. The following parameters were used for amplification, 2 min of initial denaturation at 95 °C followed by 30 cycles of amplification with a 40 s denaturation at 95 °C, 45 s of annealing at 51 °C, and 1 min of extension at 72 °C. An extra final extension step of 15 min at 72 °C was added after the completion of the 30 cycles.

### Gel electrophoresis

After the completion of the PCR amplification 10-15 μl of amplified products were used to check the amplification in 1% agarose gel casting with ethidium bromide staining in TAE buffer, at about 80 V until the marker dye reached near the end of the gel. Gels were photographed under a UV transilluminator. After the PCR and gel analysis IturinC, fenA, fenB, fenD, and srfA were detected with proper- sized amplicons in agarose gel electrophoresis with proper annealing temperature.

### Invitro bioassay

#### Fungal and pure culture maintenance

By subculturing on PDA (Potato Dextrose Agar) media and incubating at 28 °C for 5 days, then storing at 4 °C, pure cultures of *Alternaria alternata* that are separated from sunflower and maize were maintained. Gram-positive, spore-forming *B. subtilis* colonies were redistributed on Bacillus differentiation agar (BDA Agar) and incubated at 30 °C for a few days. Culture plates were maintained at 4 °C with repeated subculturing in order to confirm the growth morphology.

### Lipopeptide extraction and antifungal activity by poison food technique method

Using the poison food method, the antimicrobial activity of lipopeptide extracted from various strains was evaluated against fungal phytopathogens. For this investigation, potato dextrose agar (PDA) was used [16].

#### Preparation of starter culture and main culture

*B. subtilis *T3, T4, T5, and T6 strain starter cultures were made by adding a loop of pure *B. subtilis* cultures from a petri plate to the 100 ml of LB broth media made for the control. It was then stored overnight in an incubator cum shaker at 30 °C and 150 rpm for shaking. From the beginning culture, the major culture was created by inoculating 15 ml of each strain into 1.5 L of LB Broth (15 ml of additional glycerol/1.5ltr). The cultures were then incubated for 4 days at 30 °C and 150 rpm in an incubator cum shaker for shaking.

#### Centrifugation and acid precipitation

Supernatant i.e., cell - free extract was collected from the main culture after centrifugation in a centrifuge at 7500 rpm for 20 min. Acid precipitation was carried out by adding 2N concentrated HCl to the supernatant to lower the pH to 2 from 9. Then it was left overnight for complete precipitation at 4 °C.

#### Separation of lipopeptide layer

Chloroform and methanol were added to the acid-precipitated supernatant in a 2:1 ratio, and the mixture was agitated for 15 min in a magnetic stirrer. The mixture was placed to a layer-separating funnel and allowed to sit for 4 h. The intermediate layer, which was white and appeared semi-liquid, was then collected. For the collection of the remaining lipopeptides, the upper and lower layer was again reextracted three times in the same manner. The layer was then collected, mixed with an equivalent amount of methanol, syringe-filtered, and kept at -20 °C for further use [7].

#### Purification of lipopeptides through High Performance Liquid Chromatography (HPLC)

Antibiotics were detected and quantified by reversed-phase HPLC as follows. The filtrate described above was injected into an HPLC column [PREP-ODS C18, 20 mm (internal diameter) 25 cm (length), 15 mm particle diameter; Shimadzu, Columbia, MD, USA]. The mobile phase components were (A) 0.1% trifluoroacetic acid (TFA) in water and (B) 0.1% TFA in acetonitrile. The compounds were eluted at a flow rate of 1 ml/min) with a linear gradient of solvent B, increasing from 30 to 100%. The elution pattern was monitored at 215 nm, pooled fractions (5 ml) were collected and concentrated and results is analyzed.

#### Antifungal activity by poison food technique method

The PDA medium was made by autoclaving, and after cooling, it was combined with lipopeptides at a concentration of 10 ug/ml media with each of T3, T4, T5, and T6 separately. The mixture was then left to set. A 6 mm disc of Alternaria alternata was then placed precisely in the centre of each petri dish after 25 ml of medium had been added. As a control, a PDA plate without lipopeptide and with a fungal disc in the centre was employed. Three more PDA plates were reproduced for each treatment. All of these plates were kept in the incubator at 27 °C for 5 days before being placed in storage at 4° C. The fungal pathogens’ radial mycelial growth was observed, and the % inhibition was computed. The inhibition rate of the pathogen (IR) was calculated using the formula as follows [24].

$$\mathrm{IR }= [({\mathrm{C}}_{2}-{\mathrm{C}}_{1})/{\mathrm{C}}_{2}]\mathrm{\, x\, }100$$

Where, C_2_ is the control colony radius and C_1_ is the average radial growth of the pathogen in the presence of an antagonist.

## Results and discussion

The isolation of the genomic DNA and PCR amplification of *iturin, fengycin*, and *surfactin* genes in *B. subtilis* showed their presence. From a *B. subtilis* culture, lipopeptides were isolated, identified using HPLC, and their effectiveness was tested against the plant disease *Alternaria alternata*.

### Purification of antifungal compounds from *Bacillus subtilis* culture

Inoculating a loop-full of pure *Bacillus subtilis* culture from a Petri plate into the 100 ml of LB broth media prepared along with the control resulted in the creation of starter cultures of the T3, T4, T5, and T6 strains of the bacteria. After 4 days, the starter culture is inoculated to create the main culture, which is then centrifuged to obtain the supernatant. The supernatant is acid precipitated, combined with an equal volume of chloroform, and put to the separating funnel. The mixture is then allowed to sit for 3 to 4 h. Methanol is collected and used to dissolve the lipopeptide layer.

### PCR amplification with specific primers

**Fig. 1:**
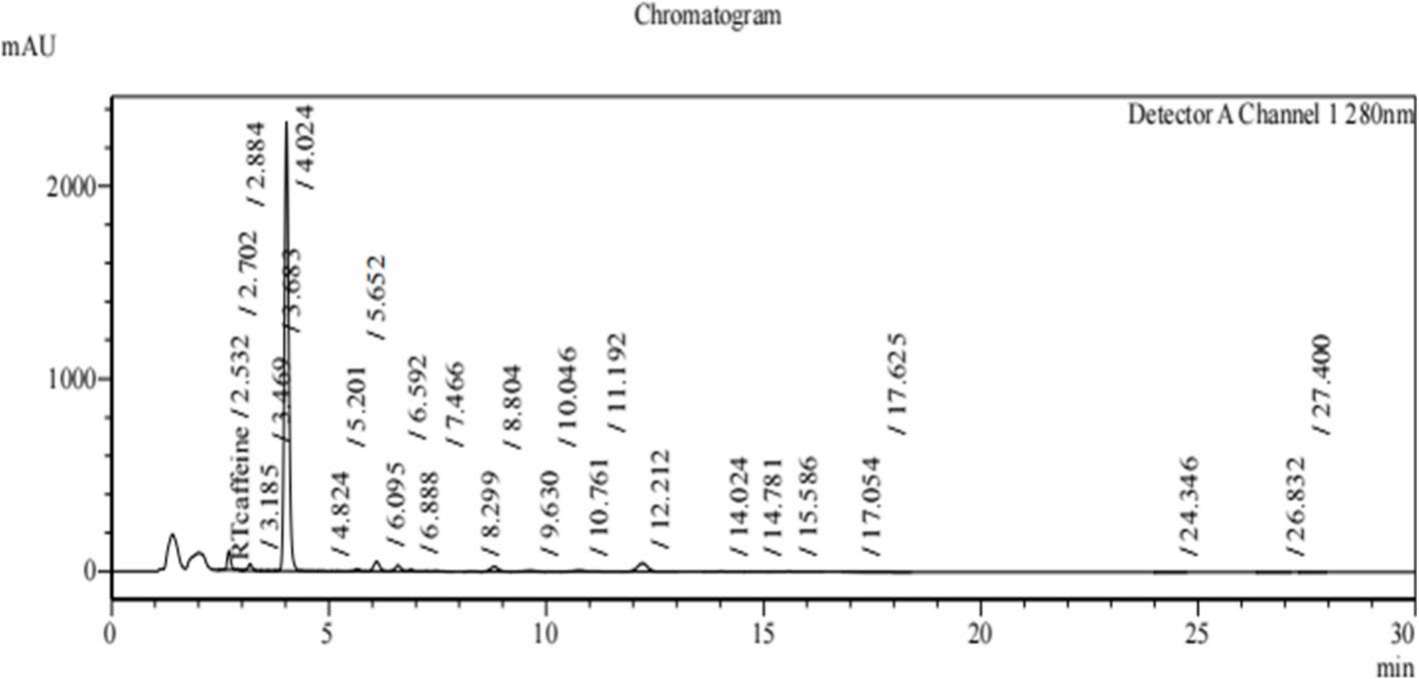
**A** *ituC* gene (594 bp), **B** *fenA* gene (820 bp), **C** *fenB* gene (670 bp), **D** *fenD* gene (269 bp) and **E** *srfA* gene (201 bp) from *B. subtilis* strains 1-T3, 2-T4,3-T5,4-T6, L-Ladder 100 bp

*B. subtilis* genomic DNA was extracted using a kit from Himedia Ltd. For validation, the purified genomic DNA was run on a 1% agarose gel, and the nano-drop reading for the DNA concentration was 63 ng/l (T3), 68.3 ng/l (T4), 86.7 ng/l (T5), and 94.8 ng/l (T6). The genes for Iturin C, fengycin A, fengycin B, fengycin D, and surfactin A were amplified using gene-specific primers. The band widths of the PCR products that were examined on an agarose gel were around 594 bp, 820 bp, 670 bp, 269 bp, and 201 bp, respectively. confirming the isolation of all the five genes used in the present study were shown in Fig. 1. In the research carried out by Meena et al. [23], observed that the gene size of the Iturin C was approximate of size 594 bp and Mota et al. [28] amplified the genes via fengycin A, fengycin B, and fengycin D in his studies and observed that they were of size approximately 800, 645 and 245 respectively. Khedher et al. [17] observed a band size of around 200 for surfactin A in their studies. All these results from previous studies are in line with the results of the present study undertaken.

### Profiling of lipopeptides extracted from *B. subtilis*

**Fig. 2:**
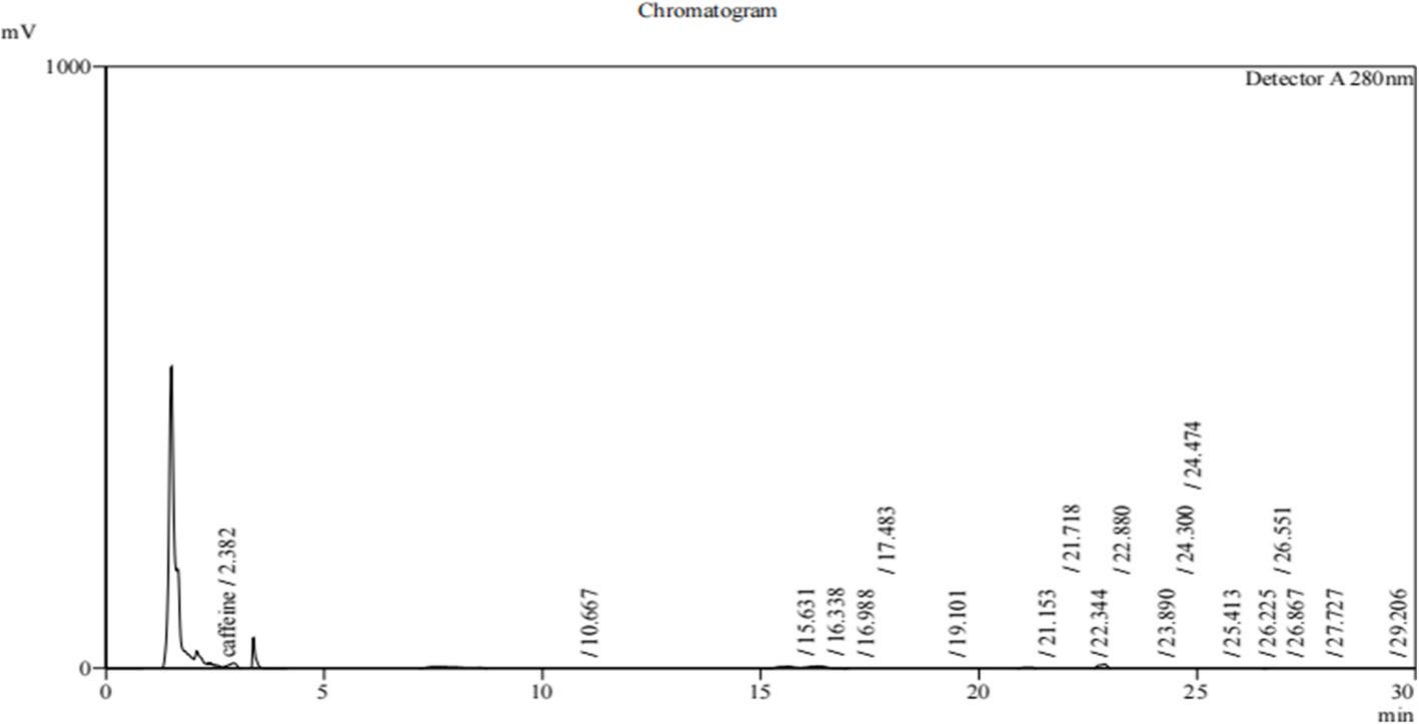
RP-HPLC profile of lipopeptide from *Bacillus subtilis* strain T3 which was subjected to RP-HPLC with gradient of mobile phases A- 0.1% trifluoroacetic- water solution and B- 0.1% (v/v) TFA-acetonitrile solution over 30 min with the detection wavelength of 280 nm

**Fig. 3:**
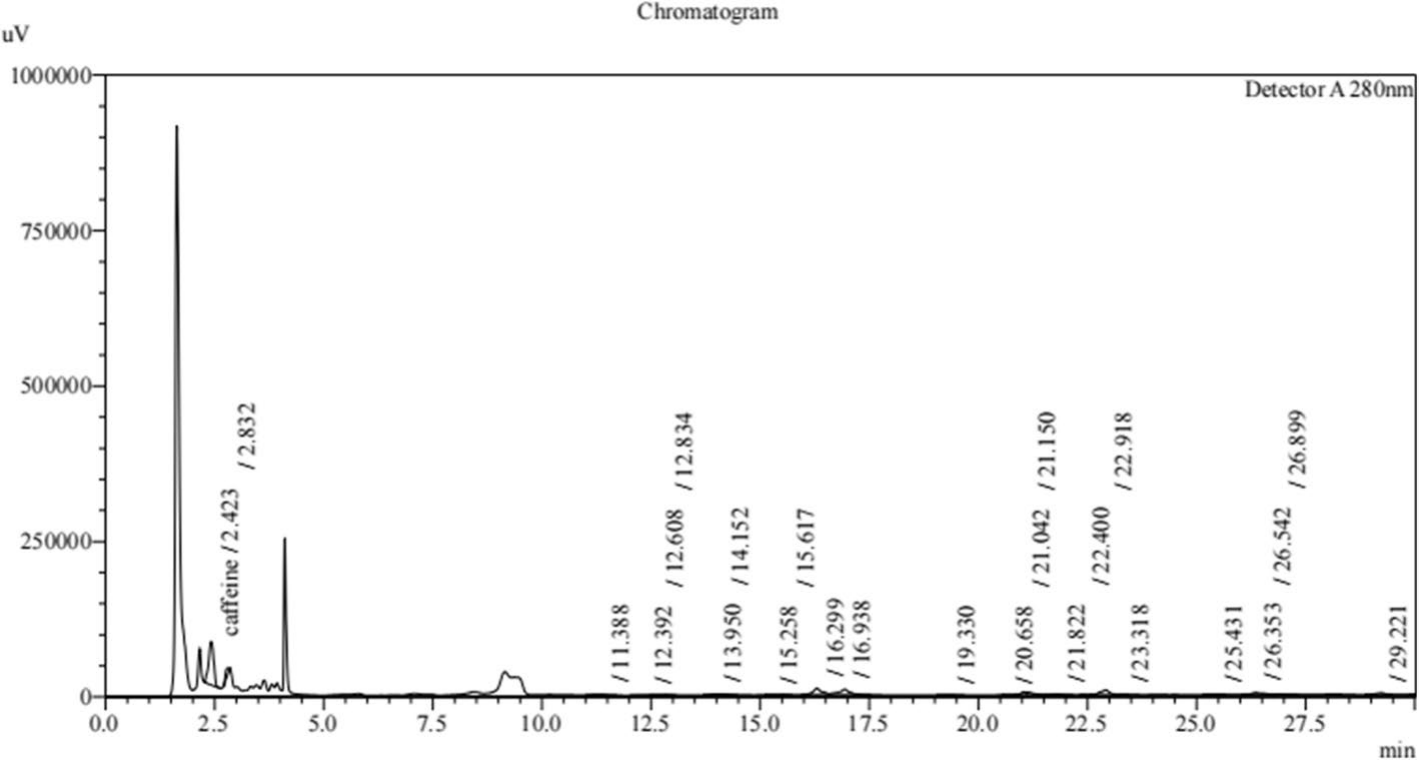
RP-HPLC profile of lipopeptide from *Bacillus subtilis* strain T4 which was subjected to RP-HPLC with gradient of mobile phases A- 0.1% trifluoroacetic- water solution and B- 0.1% (v/v) TFA-acetonitrile solution over 30 min with the detection wavelength of 280 nm

**Fig. 4:**
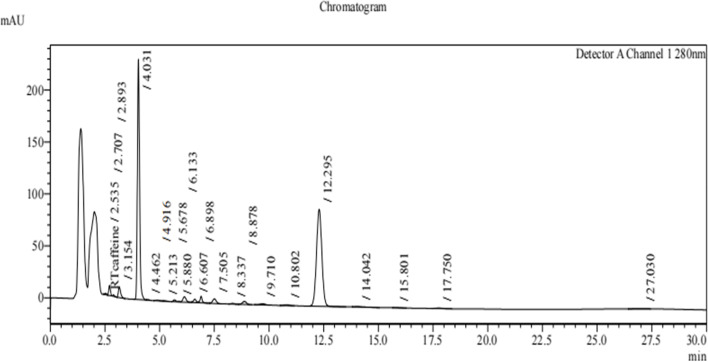
RP-HPLC profile of lipopeptide from *Bacillus subtilis* strain T5 which was subjected to RP-HPLC with gradient of mobile phases A- 0.1% trifluoroacetic- water solution and B- 0.1% (v/v) TFA-acetonitrile solution over 30 min with the detection wavelength of 280 nm

**Fig. 5:**
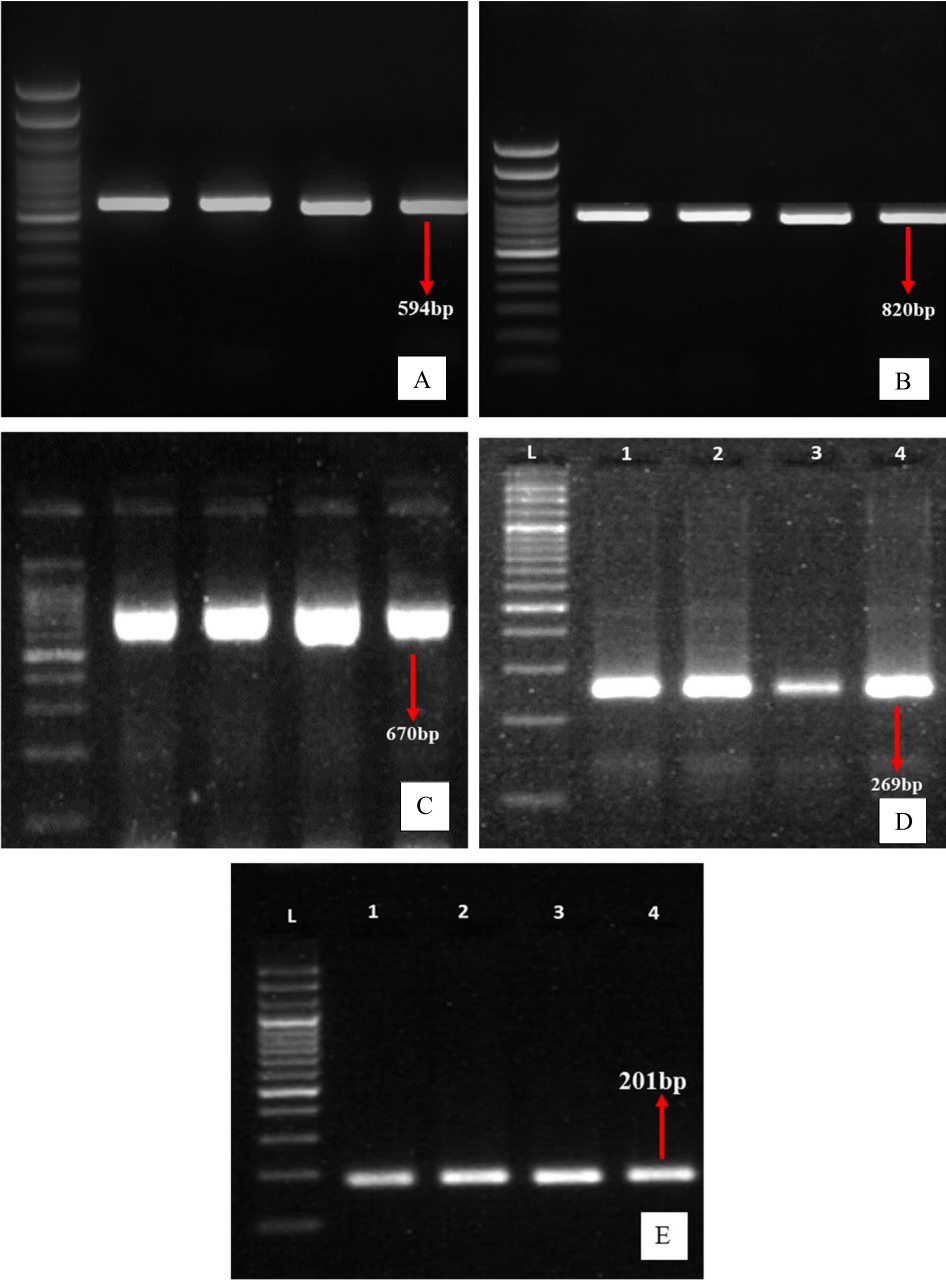
RP-HPLC profile of lipopeptide from *Bacillus subtilis* strain T6 which was subjected to RP-HPLC with gradient of mobile phases A- 0.1% trifluoroacetic- water solution and B- 0.1% (v/v) TFA-acetonitrile solution over 30 min with the detection wavelength of 280 nm

The profiling of lipopeptides extracted from *B. subtilis* strains T3, T4, T5, and T6 was carried out using Reverse Phase High - Performance Liquid Chromatography (RP-HPLC) [20, 34]. The absorbance was measured at 280 nm with a C18 column and eluted at a flow rate of 1 ml/min which is specific to detect lipopeptides. The profiles revealed multiple putative lipopeptide peaks that were present and eluted at various retention durations (Figs. 2, 3, 4 and 5). Each run uses 5 ml of sample to filter the lipopeptides produced by each strain via a nylon 0.22 micro meter syringe filter. From the HPLC Figs. 2, 3, 4 and 5 of different strains, the peaks obtained in each run show the different biological compounds (lipopeptides) present in the strains among these T6 strain showing the highest lipopeptides presence followed by T5, T3, and T4. Thus, the analysis confirms the presence of antifungal agents and these can inhibit the anti-fungal function of the *Alternaria* [32].

### In vitro bioassay by poison food techniques

**Fig. 6:**
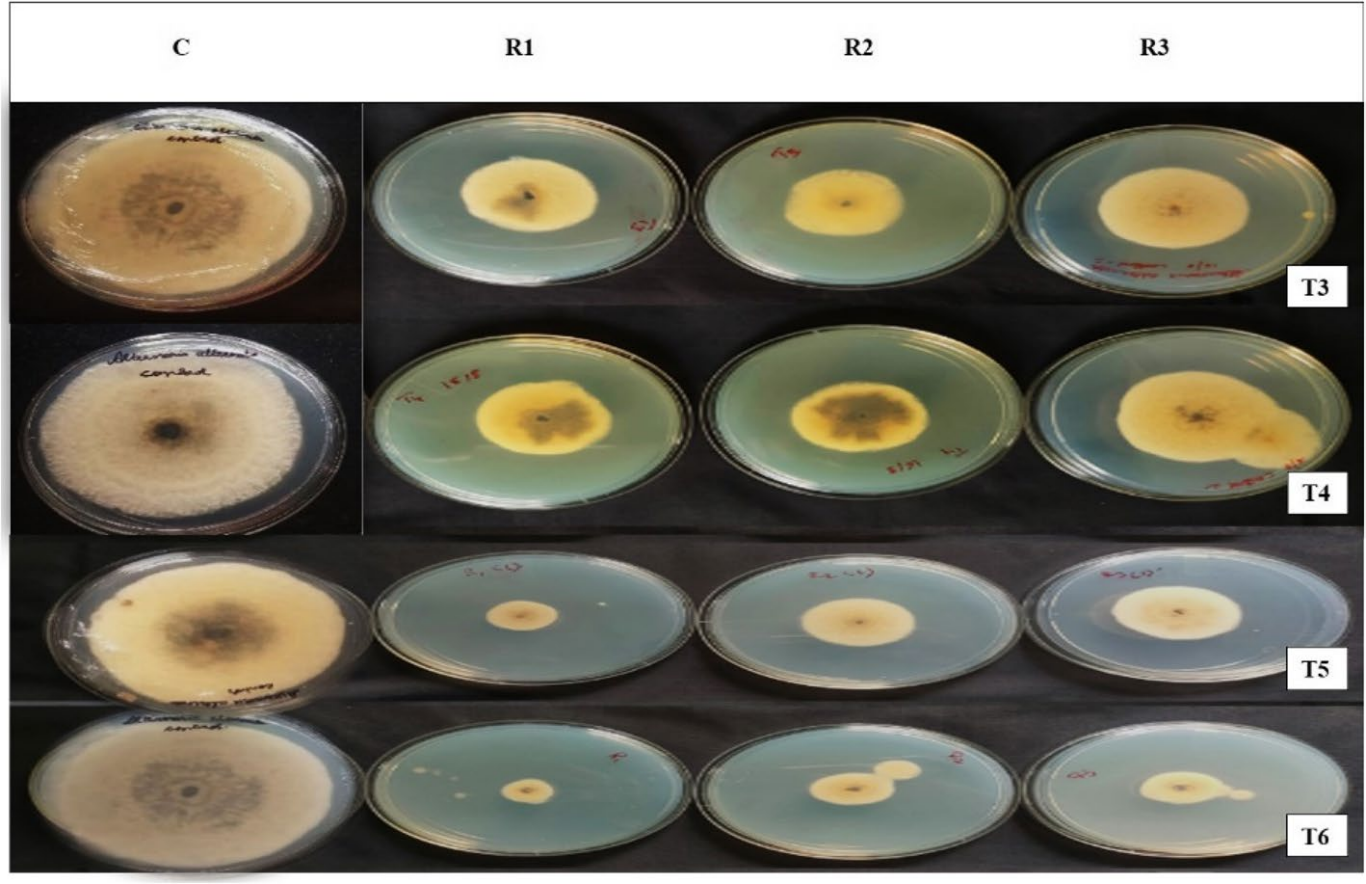
Antifungal activity of lipopeptide extract of *Bacillus subtilis* strains against *Alternaria alternate* (C. control, R1, R2, R3 Replications of T3, T4, T5 and T6 strain)

**Fig. 7:**
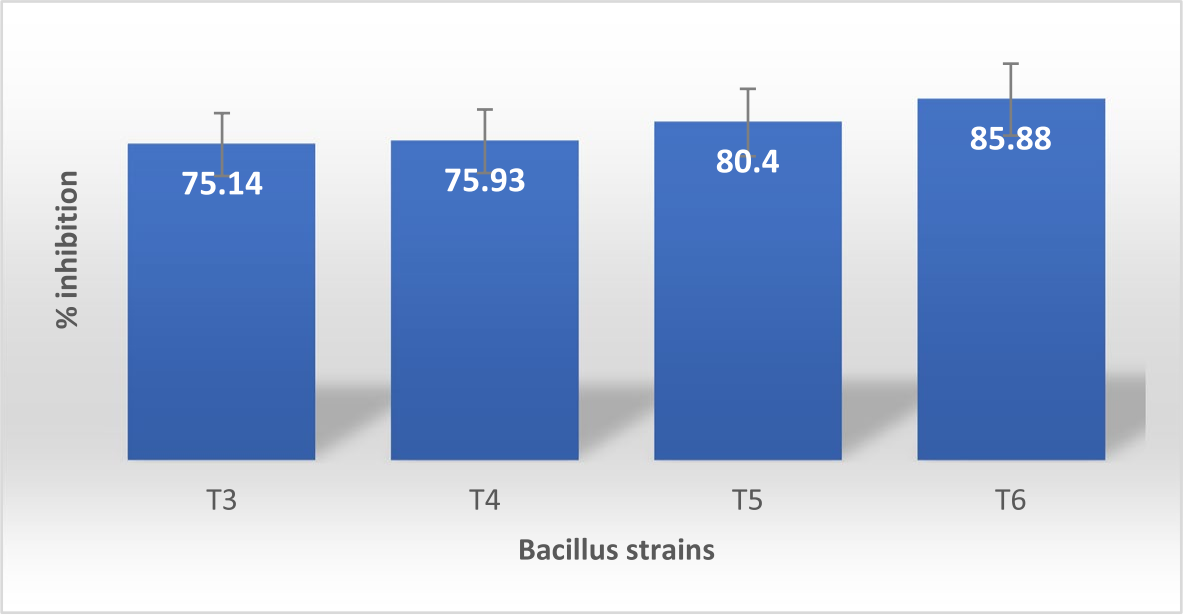
Anti-fungal activity of *Bacillus subtilis* strains (T3, T4, T5 and T6) against *Alternaria alternate*

The need for biological control of plant pathogens has significantly increased as sustainable and environmentally friendly alternatives to the massive use of fungicides [29]. The amount of extracted lipopeptide was measured, and the values for strains T3 (24 g/ml), T4 (32 g/ml), T5 (28 g/ml), and T6 (18 g/ml) were obtained. The bacterial lipopeptides have antifungal action against *Alternaria alternata*, and a comparable growth suppression zone was seen (Fig. 6) as reported by Caulier et al. [8]. A 4 mm radius in the control with a 0% inhibition rate was observed to inhibit the growth range in the T3, T4, T5, and T6 strains of *B. subtilis*. This finding was made for *Alternaria alternata*. The Poison Food Technique method revealed that the inhibitory activity of the T3, T4, T5, and T6 strains’ lipopeptides against *Alternaria alternata* was, respectively, 75.14, 75.93, 80.40, and 85.88 percent (Fig. 7), Khedher et al. [17] and Yao et al. [33] observed the same effectivity against different species and the inhibition observed on *Pythium aphanidermatum* by Kipngeno et al. [18]. According to Essghaier et al. [11], the *B. subtilis* J9 demonstrated strong growth mycelial inhibition (better than 95.3%), which inhibited the development of several phytopathogenic fungi, including *Sclerotinia, Phytophthora, Penicillium*, and *Alternaria*. The J9 strain’s biocontrol function inhibited the growth of pathogens by producing compounds that could prevent widespread illnesses and encourage plant growth.

## Conclusion

The current investigation found that the strains employed for the study had substantial antifungal activity and were effective at suppressing the plant fungal infections. When extracted lipopeptide was employed against *Alternaria*, the rate of inhibition was seen, and it was successfully managed. The T6 strain displayed the best antifungal activity against *Alternaria* among the four strains (85.88%). The in vitro results demonstrated that *B. subtilis* isolates, such as lipopeptide, have a great potential to be used as a biocontrol agent for the management of pathogens like *Alternaria*, which helps to reduce crop loss due to pathogens, as well as maintains good soil health and encourages plant growth and development. Through a variety of processes, lipopeptide-producing bacteria like *Bacillus spp*. can play a significant part in the control of plant diseases and boost agricultural output. These bacteria are some of the greatest possibilities for creating effective biopesticides due to their capacity to generate Bacillus spores. The dryness required for formulation into stable products is highly resistant to these spores. Thus, the goal of the current work is to decrease plant diseases by employing *B. subtilis* lipopeptide to regulate phytopathogens.

## Supplementary Information

**Additional file 1: Figure S1.** Original unprocessed gels identified during the lab studies.

## Data Availability

The datasets used and/or analysed during the current study available from the corresponding author on reasonable request.
