# Supplementary figures and images for: Molecular characterization and antifungal activity of lipopeptides produced from Bacillus subtilis against plant fungal pathogen Alternaria alternata
Source: BMC Microbiol. 2023 Jul 7;23:179. doi: 10.1186/s12866-023-02922-w (PMC10327374; doi:10.1186/s12866-023-02922-w)

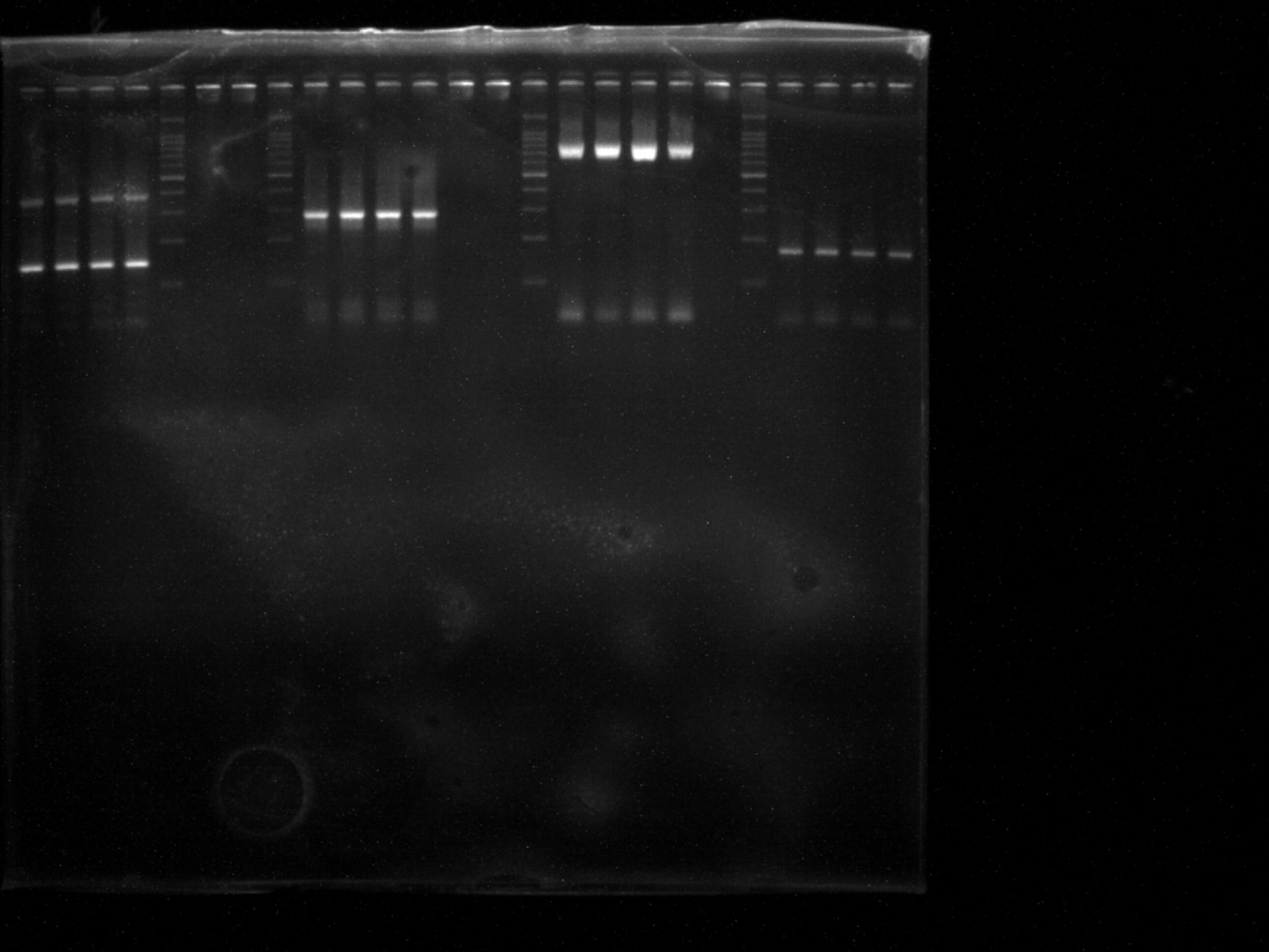


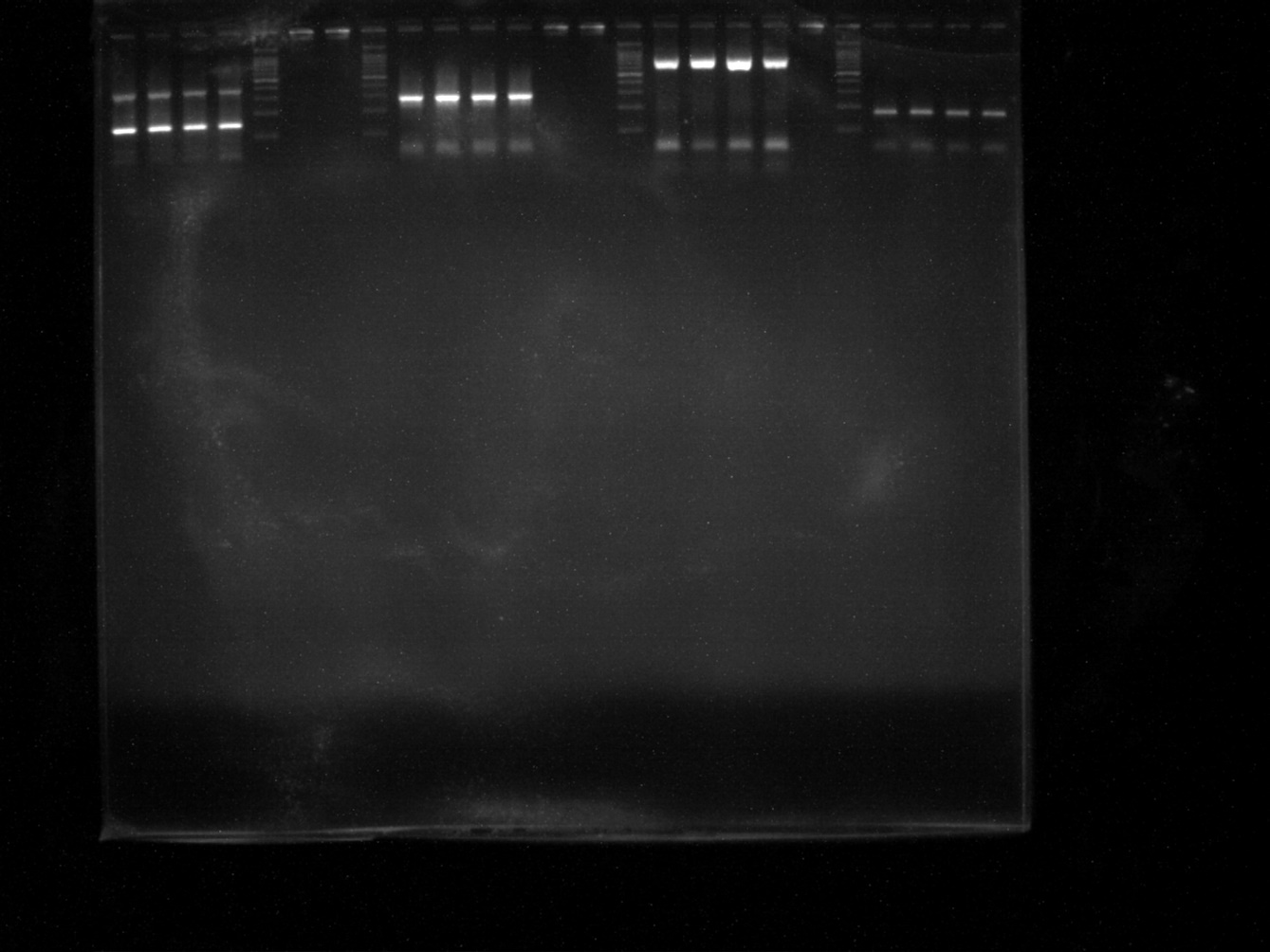


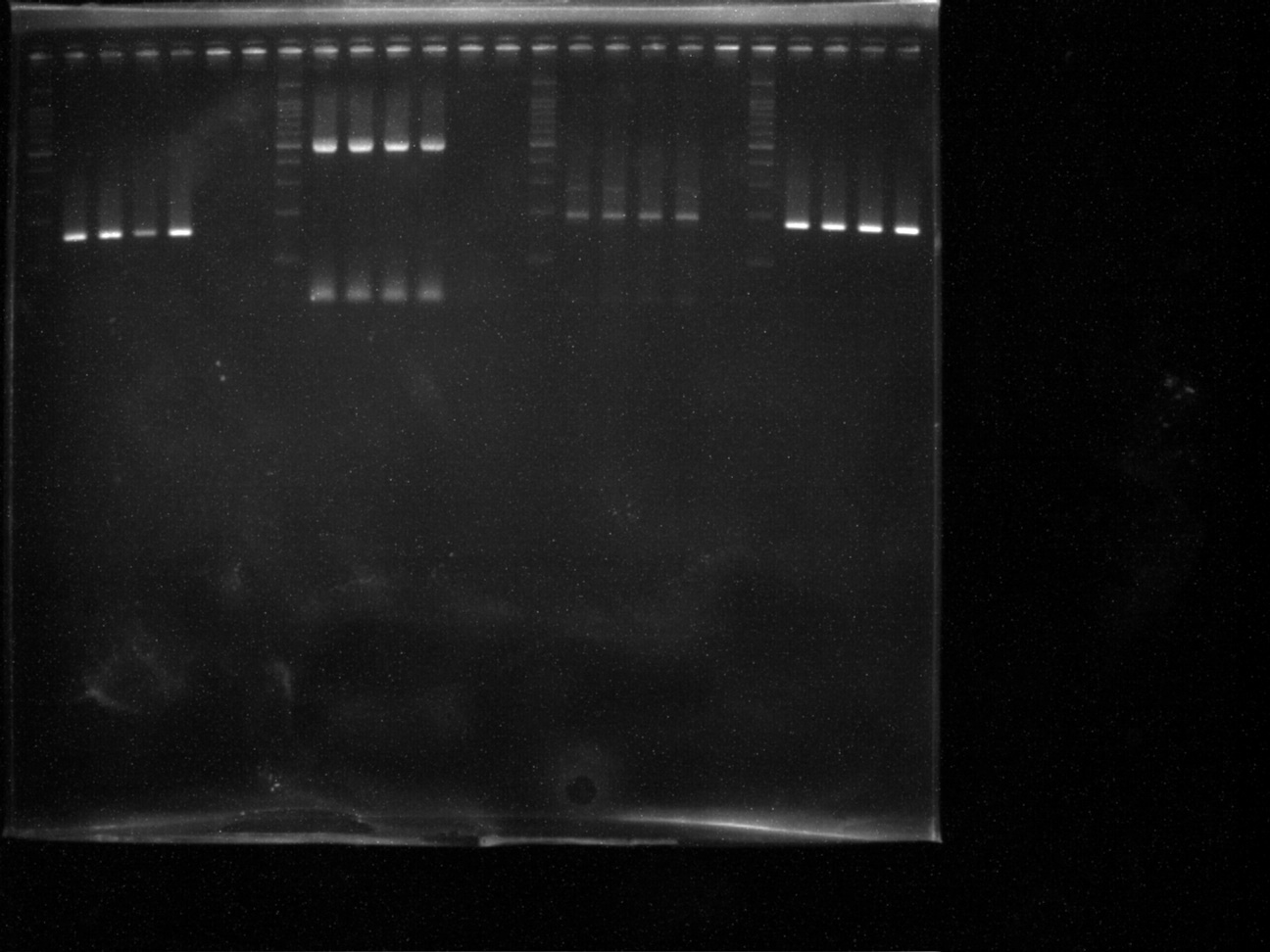


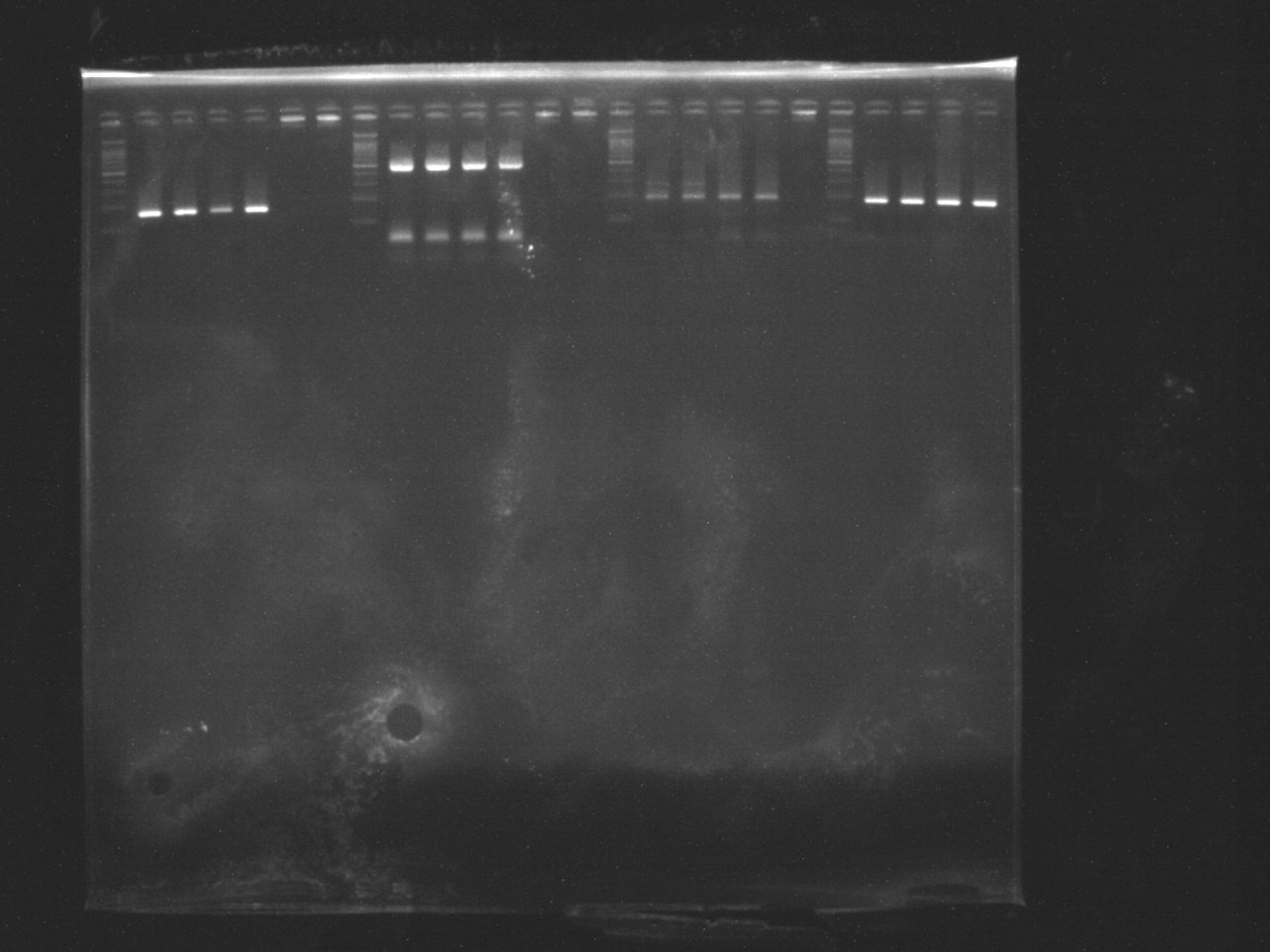


**Figure S1: Original unprocessed gels identified during the lab studies**

Supplement: Supplementary file 1 — Additional file 1: Figure S1. Original unprocessed gels identified during the lab studies. [file 12866_2023_2922_MOESM1_ESM.docx]
